# Prediction of Graft Survival Post-liver Transplantation by L-GrAFT Risk Score Model, EASE Score, MEAF Scoring, and EAD

**DOI:** 10.3389/fsurg.2021.753056

**Published:** 2021-11-19

**Authors:** Shirui Chen, Tielong Wang, Tao Luo, Shujiao He, Changjun Huang, Zehua Jia, Liqiang Zhan, Dongping Wang, Xiaofeng Zhu, Zhiyong Guo, Xiaoshun He

**Affiliations:** ^1^Organ Transplant Center, The First Affiliated Hospital, Sun Yat-sen University, Guangzhou, China; ^2^Guangdong Provincial Key Laboratory of Organ Donation and Transplant Immunology, Guangzhou, China; ^3^Guangdong Provincial International Cooperation Base of Science and Technology, Guangzhou, China

**Keywords:** orthotopic liver transplantation, risk prediction model, machine perfusion, risk factor, graft survival, patient survivability

## Abstract

**Background:** Early allograft dysfunction (EAD) is correlated with poor patient or graft survival in liver transplantation. However, the power of distinct definitions of EAD in prediction of graft survival is unclear.

**Methods:** This retrospective, single-center study reviewed data of 677 recipients undergoing orthotopic liver transplant between July 2015 and June 2020. The following EAD definitions were compared: liver graft assessment following transplantation (L-GrAFT) risk score model, early allograft failure simplified estimation score (EASE), model for early allograft function (MEAF) scoring, and Olthoff criteria. Risk factors for L-GrAFT_7_ high risk group were evaluated with univariate and multivariable logistic regression analysis.

**Results:** L-GrAFT_7_ had a satisfied C-statistic of 0.87 in predicting a 3-month graft survival which significantly outperformed MEAF (C-statistic = 0.78, *P* = 0.01) and EAD (C-statistic = 0.75, *P* < 0.001), respectively. L-GrAFT_10_, EASE was similar to L-GrAFT_7_, and they had no statistical significance in predicting survival. Laboratory model for end-stage liver disease score and cold ischemia time are risk factors of L-GrAFT_7_ high-risk group.

**Conclusion:** L-GrAFT_7_ risk score is capable for better predicting the 3-month graft survival than the MEAF and EAD in a Chinese cohort, which might standardize assessment of early graft function and serve as a surrogate endpoint in clinical trial.

## Introduction

Orthotopic liver transplantation (OLT) has been accepted as the treatment of choice for patients with end-stage liver disease (1). However, due to the reality of increasing organ shortage, use of marginal livers is considered as an effective method to expand donor pool (2–4). However, transplantation of these livers are associated with increased the incidence of poor allograft function after the OLT at the same time (5–8).

Early allograft dysfunction (EAD) is proposed to describe initial poor graft function, (9) and the impact of EAD after OLT is associated with poor patient and allograft survival among centers (10–12). However, the specific definition of EAD is still controversial. Deschênes et al. firstly used serum bilirubin, prothrombin time and hepatic encephalopathy, which are considered as the basic parameters to define EAD (9). After modifications, in the model for end-stage liver disease (MELD) era, Olthoff et al. created the most widely used definition of EAD, which was defined as meeting one or more of the following conditions: (1) bilirubin ≥10 mg/dL on post-operation day (POD) 7, (2) international normalized ratio (INR) ≥1.6 on POD 7, and (3) alanine aminotransferases (ALT) or aspartate aminotransferases (AST) >2,000 IU/L within the first 7 days (12).

However, the dichotomous outcome could not accurately grade early hepatic allograft function. In that case, Pareja et al. created a continuous score model named model for early allograft function (MEAF) scoring to evaluate EAD (13). The MEAF score consists of 3 scores related to post-operative laboratory analyses: the maximum of ALT and INR within the first 3 days and the bilirubin on POD 3. However, the MEAF does not take the changes and trend of the laboratory test into account, which might mistake patients getting better from those getting worse. Recently, Agopian et al. created a new continuous score model called liver graft assessment following transplantation (L-GrAFT) risk score, which is using 7- or 10-days post-operative laboratory variables [ALT, INR, total bilirubin (TBIL), and platelet (PLT)] to calculate risk score and evaluate the graft failure risk (14, 15). Moreover, Avolio et al. created a comprehensive model named the early allograft failure simplified estimation (EASE) score to evaluate early allograft failure. What they though were taking MELD, transfusion, post-operative thrombosis of a hepatic vessel, and center volume into account (16).

In this study, we want to validate these models' efficacy for assessing graft function after OLT in our center. At the same time, we want to determine which model is a better indicator of graft outcome and analyze the related risk factors.

## Patients and Methods

### Study Design and Patient Selection

Of the 827 cases, 821 recipients who underwent an OLT at The First Affiliated Hospital of Sun Yat-sen University from July 1, 2015 to June 30, 2020 were included in this single-center retrospective study. The recipient exclusion criteria were recipient age <18 years, OLT for acute liver failure, retransplantation, multivisceral transplantation, split liver transplantation, living donor transplantation, early post-transplant vascular complications (<14 days), and insufficient data for model score calculation. No organs from executed prisoners were used.

Data collection began on July 15, 2020 and ended on October 31, 2020. Data analysis began on November 1, 2020 and ended on December 31, 2020. This study was conformed to the ethical guidelines of the 1975 Declaration of Helsinki and approved by Independent Ethics committee for Clinical Research and Animal Trails of The First Affiliated Hospital of Sun Yat-sen University (No. [2020]336).

The criteria of EAD defined by Olthoff et al. is the presence of one or more variables (12), such as: (1) bilirubin ≥10 mg/dL on POD 7, (2) INR ≥1.6 on POD 7, and (3) ALT or AST >2,000 IU/L within the first 7 days.

The MEAF score can be calculated as follows (13): MEAF = (score ALT_max.3POD_ + score INR_max.3POD_ + score bilirubin_3POD_), score ALT_max.3POD_ = 3.29/{1 + e^−1.9132[ln(ALTmax.3POD)−6.1723]^}, score INR_max.3POD_ = 3.29/{1 + e^−6.8204[ln(INRmax.3POD)−0.6658]^}, score bilirubin_3POD_ = 3.4/{1 + e^−1.8005[ln(bilirubin3POD)−1.0607]^}. Five MEAF risk groups for graft and patient survival were defined as follows: (1) risk group 1: 0<MEAF score≤2; (2) risk group 2: 2<MEAF score≤5; (3) risk group 3: 5<MEAF score≤6; (4) risk group 4: 6<MEAF score≤8; (5) risk group 5: 8<MEAF score≤10.

Liver graft assessment following transplantation risk score uses 7 (L-GrAFT_7_) or 10 (L-GrAFT_10_) days' post-operative laboratory variables to evaluate, and measures the average by area under curve (AUC) (17): L-GrAFT_7_ = 6.9647 – 0.5799^*^(AUC ln AST) + 0.00844^*^(AUC ln AST)^2^ + 5.25347^*^(slope ln AST) + 4.65046^*^(slope ln AST)^2^ + 1.14098^*^(ln AUC INR) – 0.03475^*^(AUC ln TBIL) + 0.00562^*^(AUC ln TBIL)^2^ + 4.31135^*^(slope ln TBIL) + 5.84724^*^(slope ln TBIL)^2^ – 0.05115^*^(AUC ln PLT), and L-GrAFT_10_ = 9.77 – 0.42926^*^(AUC ln AST) + 0.00462^*^(AUC ln AST)^2^ + 4.60719^*^(Slope_7_ ln AST) + 4.4129^*^(Slope_7_ ln AST)^2^ + 0.88974^*^(ln max INR) – 0.04852^*^(AUC ln TBIL) + 0.00363^*^(AUC ln TBIL)^2^ + 5.33627^*^(slope ln TBIL) – 0.04621^*^(AUC ln PLT) – 5.24897^*^(slope ln PLT) + 13.08633^*^(slope ln PLT)^2^. Seven L-GrAFT_7_ risk groups for graft and patient survival were defined as follows: (1) risk group 1: L-GrAFT_7_ score<-3.5; (2) risk group 2: −3.5≤L-GrAFT_7_ score<-2.5; (3) risk group 3: −2.5≤L-GrAFT_7_ score<-1.5; (4) risk group 4: −1.5≤L-GrAFT_7_ score<-0.5; (5) risk group 5: −0.5≤L-GrAFT_7_ score <0.5; (6) risk group 6: 0.5≤L-GrAFT_7_ score<1.5; (7) risk group 7: 1.5≤L-GrAFT_7_ score<7.5. L-GrAFT_7_ low risk groups contain risk groups 1–3 and high risk groups contain risk groups 4–7. Five L-GrAFT_10_ risk groups for graft and patient survival were defined as follows: (1) risk group 1: L-GrAFT_10_ score<-3.23; (2) risk group 2: −3.23≤L-GrAFT_10_ score<-1.18; (3) risk group 3: −1.18≤L-GrAFT_10_ score<-0.57; (4) risk group 4: −0.57≤L-GrAFT_10_ score<1.3; (5) risk group 5: 1.3≤L-GrAFT_10_ score.

EASE score's formula is as follows (16): EASE = −0.602 + 0.044^*^(MELD at transplant) + 0.065^*^(number of PACKED RED BLOOD CELL transfused units during surgery) + 2.567 (if arterial or portal thrombosis during days 1–10) + 0.000534^*^(AUC ln AST in POD1,2,3,7,10)^2^ – 0.093^*^(AUC ln PLT in POD1,3,7,10) – 7.766^*^(slope ln PLT in POD1,3,7,10) + 0.795^*^(slope ln billirubin in POD1,3,7,10) – 0.402 (if center volume ≥70 cases per year). Five EASE risk groups for graft and patient survival were defined as follows: (1) risk group 1: EASE score < −3.43; (2) risk group 2: −3.43 ≤ EASE score ≤ −1.26; (3) risk group 3: −1.25 ≤ EASE score ≤ −0.75; (4) risk group 4: −0.74 ≤ EASE score ≤ −0.01 (5) risk group 5: 0 ≤ EASE score ≤ 5.

### Statistical Analysis

Continuous variables are reported as median values (interquartile range) and categorical variables as percentage. Patients without need for a retransplantation or death was counted for the calculation of graft survival. Patient survival and graft survival curves were constructed using Kaplan-Meier method and compared using log-rank tests. The receiver operating characteristic (ROC) analysis was used to evaluate model accuracy, and the area under the ROC curves (AUROC) were compared among L-GrAFT_7_, L-GrAFT_10_, EASE, MEAF, and EAD. By using the Delong et al. method, the AUROCs were compared between each pair of definitions (18). Recipient, donor, and operation-related risk factors were compared between L-GrAFT_7_ high-risk groups and low-risk groups using the non-conditional univariate logistic regression analysis. After excluding variables with potential multicollinearity, we entered the rest, whose *P* < 0.1 in the univariate logistic regression analysis, into a multivariable logistic regression model.

Analyses were performed using SPSS (version 26.0), MedCalc (version 19.5.3) and GraphPad Prism (version 8.4.0).

## Results

During the study period, 827 liver transplants were performed, and 150 cases were excluded due to the following reasons: recipient age < 18 years (17 cases), OLT for acute liver failure (13 cases), retransplantation (10 cases), multivisceral transplantation (31 cases), split liver transplantation (12 cases), living donor transplantation (one case), early vascular complications (23 cases), data not available (12 cases), and not enough laboratory values (31 cases). Then 677 cases remained to be analyzed in this study. The recipients, donor, and operation-related characteristics are shown in Supplementary Table 1. Since data for some variables were not available for all cases, the results presented were based on available information only.

The median age of recipients was 51 years and 88.8% were men. Hepatocellular carcinoma (HCC) accounted for 53.3% of all recipients' primary reason for OLT and was followed by Hepatitis B virus-related cirrhosis (22.0%). Comorbidities like diabetes, hypertension and cardiovascular system disease were presented in 14.8, 13.6, and 3.5% cases, respectively. The median laboratory MELD score in all cases was 12 whereas in HCC and non-HCC cases it was 8 or 18, respectively.

With regard to the donors, the median age was 38 years, and 74.9% were men. Trauma was the first reason (47.7%) that causes death of donors, and the second was cerebrovascular accident (37.2%). According to the Chinese classification of deceased organ donation (19), 79.3%, 16.2%, and 4.4% organs were from donation after brain death (DBD), donation after cardiac death (DCD), and donation after brain and cardiac death (DBCD), respectively.

In this study, the OLT operations were performed with a median cold ischemia time (CIT) of 421 min and a median total operation time of 450 min. The median red blood cell transfusion was 5.0 units, and intensive care unit (ICU) stay was 40.0 h. With a median follow-up time of 19.57 months, the overall patient survival was 93.8, 92.5, and 89.1% at 3-, 6-, and 12 months, respectively, and the overall graft survival were 93.6, 92.3, and 89.0% at 3-, 6-, and 12 months, respectively (Supplementary Figure 1).

### Prediction Power of L-GrAFT Models vs. EASE, MEAF, and EAD

**Figure 1:**
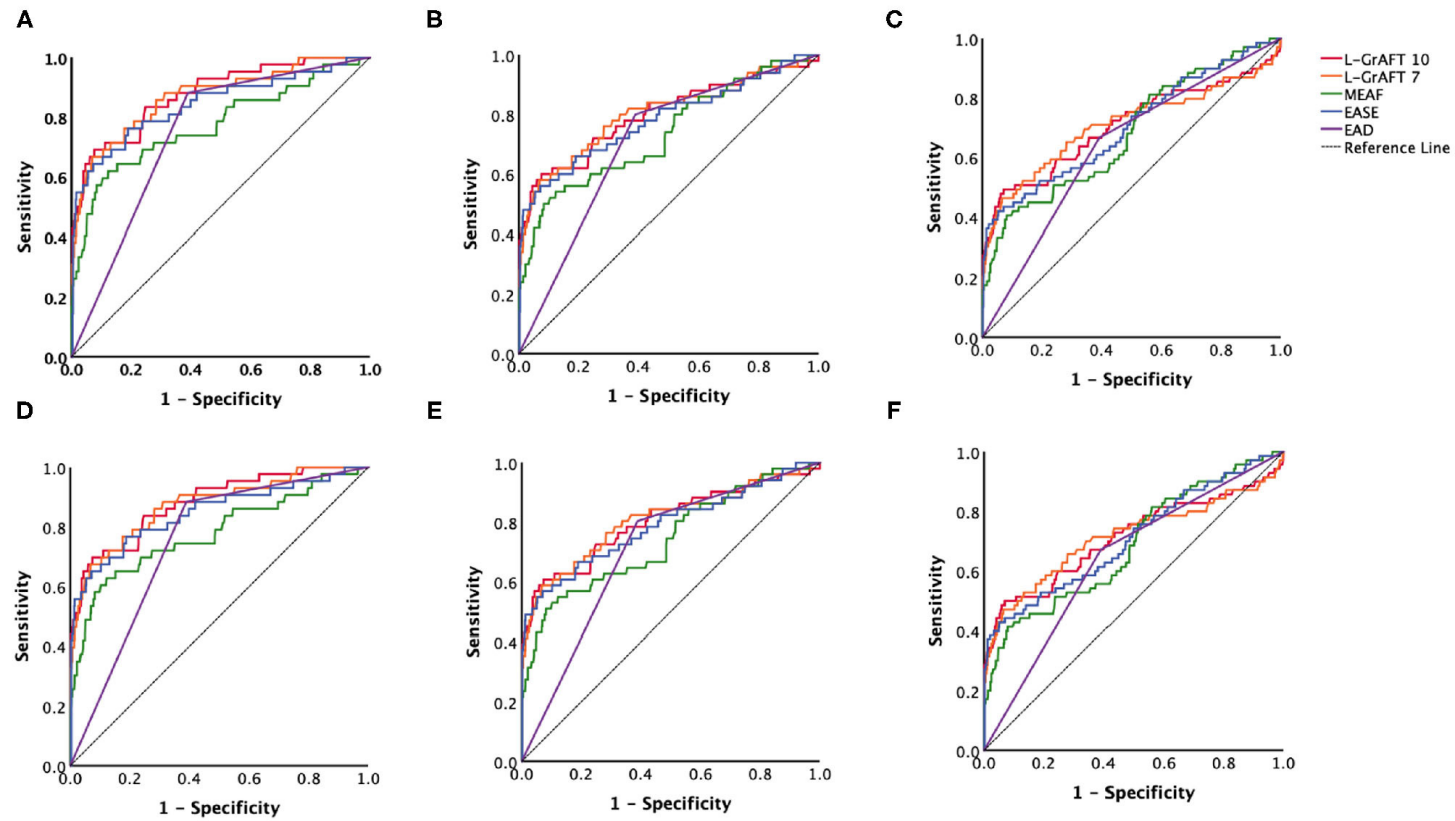
Models accuracies compared by the area under the receiver operating characteristic (AUROC). AUROC curves comparison among L-GrAFT_10_, L-GrAF_7_, EASE, MEAF, and EAD for predicting **(A)** 3-month patient survival, **(B)** 6-month patient survival, **(C)** 12-month patient survival, **(D)** 3-month graft survival, **(E)** 6-month graft survival, and **(F)** 12-month graft survival. Specific AUROC data are shown in Table 1.

**Figure 2:**
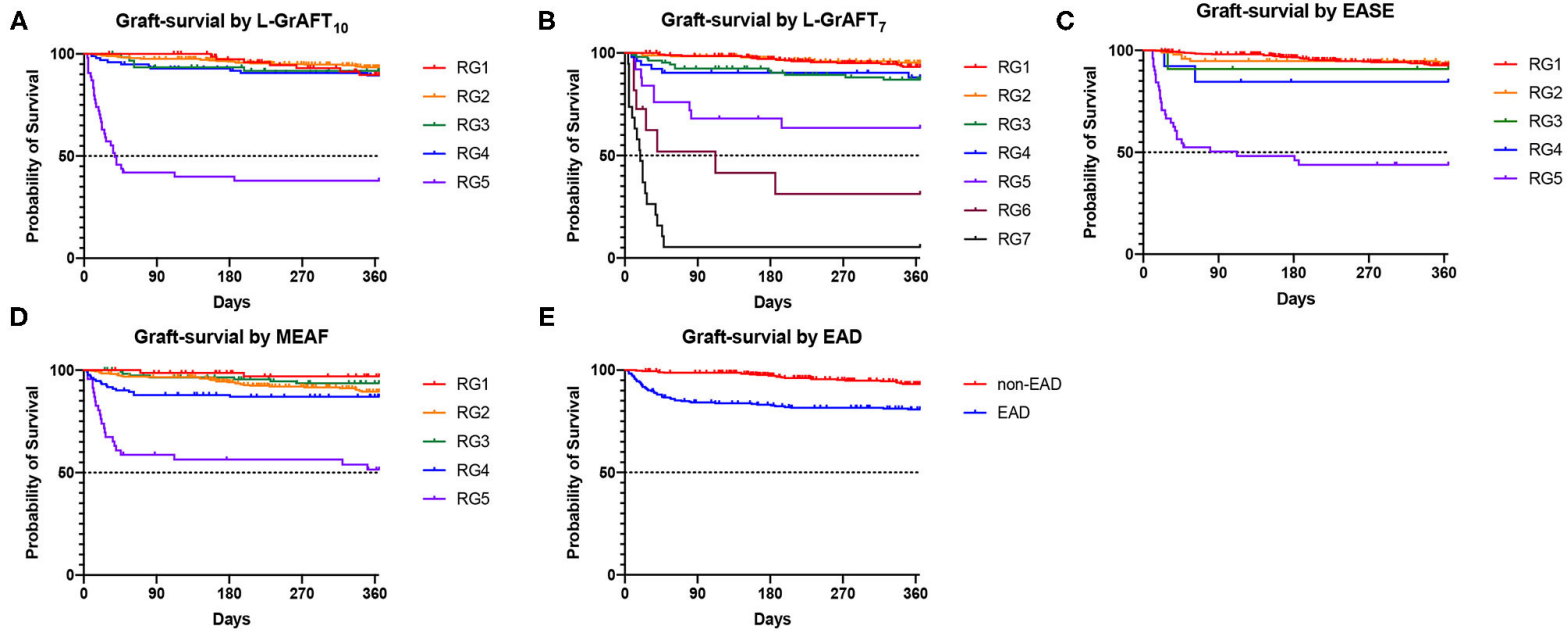
Graft survival according to the L-GrAFT_10_, L-GrAFT_7_, EASE, MEAF, and EAD risk classes. **(A)** Graft-survival by L-GrAFT_10_; **(B)** Graft-survival by L-GrAFT_7_; **(C)** Graft-survival by EASE; **(D)** Graft-survival by MEAF; **(E)** Graft-survival by EAD. EAD, early allograft dysfunction; EASE, Early Allograft Failure Simplified Estimation; L-GrAFT, Liver Graft Assessment Following Transplantation; MEAF, Model for Early Allograft Function Scoring; RG, risk group.

Based on the results shown in Figure 1, Table 1, both L-GrAFT_10_ and L-GrAFT_7_ had good AUROC of 0.88 and 0.87 in predicting the 3-month graft survival which significantly outperformed MEAF score model (AUROC = 0.77, vs. L-GrAFT_10_ *P* = 0.01, vs. L-GrAFT_7_ *P* = 0.01) and EAD (AUROC = 0.75, vs. L-GrAFT_10_ *P* < 0.001, vs. L-GrAFT_7_ *P* < 0.001), respectively. EASE had a AUROC of 0.84 in predicting 3-month graft survival and outperform EAD (*P* = 0.01). When it came to predicting 6-month or 12-month graft survival, all models have a smaller AUROC and the difference between L-GrAFT, EASE, and MEAF is milder. In predicting the 3-, 6-, and 12-month graft survival, L-GrAFT_10_, L-GrAFT_7_, and EASE were significantly better than EAD all the time. The AUROCs among L-GrAFT_10_, L-GrAFT_7_, and EASE (data not shown) and the AUROCs between MEAF and EAD failed to obtain a statistical significance in predicting 3-, 6-, and 12-month graft survival, respectively. The AUROCs and comparation among models in predicting patient survival had a similar result. Besides, L-GrAFT_7_ had a good ability to differentiate relevant risk among risk groups in both graft survival and patient survival than L-GrAFT_10_, MEAF, EASE, and EAD (Figure 2; Supplementary Figure 2).

**Table 1:** Validation AUROC and comparison among L-GrAFT_10_, L-GrAFT_7_, EASE, MEAF, and EAD.

	**AUROC (95% CI)**					**L-GrAFT_**10**_ vs. MEAF**	**L-GrAFT_**7**_ vs. MEAF**	**L-GrAFT_**10**_ vs. EAD**	**L-GrAFT_**7**_ vs. EAD**	**EASE vs. EAD**
	**L-GrAFT_**10**_**	**L-GrAFT_**7**_**	**EASE**	**MEAF**	**EAD**	***P*-value**	***P*-value**	***P*-value**	***P*-value**	***P*-value**
**ALL PATIENTS**										
**Patient survival**										
3 month	0.88 (0.82–0.94)	0.87 (0.81–0.93)	0.84 (0.77–0.92)	0.77 (0.69–0.86)	0.75 (0.68–0.81)	0.01	0.01	<0.001	<0.001	0.01
6 month	0.80 (0.72–0.88)	0.80 (0.72–0.88)	0.78 (0.70–0.86)	0.74 (0.65–0.82)	0.71 (0.64–0.78)	0.16	0.07	0.005	0.001	0.03
12 month	0.71 (0.63–0.79)	0.71 (0.63–0.79)	0.70 (0.63–0.78)	0.68 (0.61–0.75)	0.64 (0.57–0.71)	0.50	0.44	0.02	0.02	0.03
**Graft survival**										
3 month	0.88 (0.82–0.94)	0.87 (0.81–0.94)	0.85 (0.77–0.92)	0.78 (0.69–0.87)	0.75 (0.68–0.81)	0.01	0.01	<0.001	<0.001	0.009
6 month	0.80 (0.73–0.88)	0.81 (0.73–0.88)	0.79 (0.71–0.87)	0.74 (0.66–0.82)	0.71 (0.64–0.78)	0.15	0.07	0.003	<0.001	0.02
12 month	0.71 (0.63–0.79)	0.71 (0.63–0.80)	0.71 (0.64–0.78)	0.69 (0.62–0.76)	0.64 (0.57–0.71)	0.49	0.44	0.01	0.02	0.02

### Risk Factors for L-GrAFT_7_ High Risk Group

Following to the results above, we decided to determine the risk factors between L-GrAFT_7_ high-risk group and low-risk group. As shown in Table 2, 150 cases were in the high-risk group and 527 cases belonged to low-risk cases. Laboratory MELD score (*P* = 0.02), TBIL (*P* = 0.01), INR (*P* = 0.03), and CIT (*P* = 0.001) had a statistical significance in univariable analysis. Using variables with *P* < 0.1 in univariable analysis, laboratory MELD score (*OR* = 1.020, *P* = 0.017) and CIT (*OR* = 1.002, *P* = 0.001) were significantly associated with L-GrAFT_7_ high-risk group (Table 3).

**Table 2:** Univariable analysis of characteristics between L-GrAFT_7_ high and low risk group.

**Variables**	**Frequency (%) or Median (IQR)**		***P*-value**
	**High-risk group**	**Low-risk group**	
	***N* = 150**	***N* = 527**	
**Recipient**			
Age (years)	51 (41, 58)	51 (43, 59)	0.09
Male	92.7	87.7	0.17
BMI (kg/m^2^)	23.5 (21.1, 25.5)	23.0 (20.6, 24.7)	0.18
Height (cm)	170.0 (165.0, 172.0)	169.0 (165.0, 172.0)	0.17
Weight (kg)	67.5 (60.0, 74.0)	65.0 (57.0, 70.0)	0.09
Diagnosis			0.10
HCC	51.3	53.9	
Hepatitis B virus-related	19.3	22.8	
cirrhosis			
ACLF	14.7	8.3	
Alcoholic cirrhosis	6.0	3.6	
Other	8.7	11.4	
Comorbidity			
Diabetes	18.0	13.9	0.21
Hypertension	15.3	13.1	0.48
Cardiovascular system	4.0	3.4	0.73
diseases			
**Pre-transplantation**			
Laboratory MELD score	13 (7, 26)	11 (6, 20)	0.02
CREA (μmol/L)	72 (60, 93)	71 (59, 88)	0.94
TBIL (μmol/L)	58.1 (26.6, 352.4)	39.5 (19.4, 178.2)	0.01
INR	1.40 (1.18, 2.15)	1.33 (1.11, 1.85)	0.03
Child-Pugh score	9 (7, 10)	8 (6, 10)	0.34
Infection	9.3	10.8	0.60
Renal replacement therapy	0.7	1.5	0.99
Mechanical ventilation	0	1.3	0.52
**Donor**			
Age (Years)	40 (26, 47)	38 (23, 47)	0.37
Male	74.7	75.0	0.94
BMI (kg/m^2^)	22.5 (20.5, 24.2)	22.0 (20.3, 23.9)	0.15
Height (cm)	168.0 (160.0, 170.0)	168.0 (160.0, 170.0)	0.82
Weight (kg)	62.5 (55.0, 70.0)	60.0 (55.0, 67.0)	0.17
Cause of death			0.63
Trauma	50.0	47.1	
CVA	34.0	38.1	
HIE	7.3	8.3	
Others	8.7	6.5	
The Chinese Classification of Deceased Organ Donation			0.18
C-I (DBD)	75.3	81.0	
C-II (DCD)	18.0	15.4	
C-III (DBCD)	6.7	3.6	
Comorbidity			
Hypertension	12.2	9.5	0.34
Diabetes	8.8	9.5	0.79
Cardiovascular system	2.0	0.6	0.12
diseases			
Hepatic steatosis			
DRI	1.72 (1.45, 2.10)	1.62 (1.41, 2.04)	0.11
**Operation-related**			
Anhepatic phase (min)	55 (45, 67)	54 (43, 64)	0.40
CIT (min)	458 (361, 565)	407 (329, 508)	0.001
Total operation time	475 (398, 540)	445 (387, 510)	0.06
Transfusion			
RBC (unit)	4.3 (2.0, 9.0)	5.0 (3.0, 8.0)	0.40
Fresh frozen plasma (unit)	8.0 (5.0, 11.3)	7.5 (5.0, 10.0)	0.81
Intraoperative hemorrhage (ml)	1500 (800, 3000)	1500 (1000, 2800)	0.28

*ACLF, acute-on-chronic liver failure; ALT, alanine aminotransferase; AST, aspartate aminotransferase; BMI, body mass index; CIT, cold ischemia time; CREA, creatinine; CVA, cerebrovascular accident; DBCD, donation after brain and cardiac death; DBD, donation after brain death; DCD, donation after cardiac death; DRI, donor risk index; HCC, hepatocellular carcinoma; HIE, hypoxic-ischemic encephalopathy; INR, international normalized ratio; MELD, Model for End-Stage Liver Disease; PLT, platelet; RBC, red blood cell; TBIL, total bilirubin*.

*Data for some variables were not available for some recipients or donors, so the results presented are based on available information only*.

**Table 3:** Multivariable analysis of characteristics between L-GrAFT_7_ high and low risk group.

**Variables**	**OR**	**95% CI**	***P*-value**
Laboratory MELD score	1.020	1.004, 1.039	0.017
CIT	1.002	1.001, 1.003	0.001

*CI, confidence interval; CIT, cold ischemia time; MELD, Model for End-Stage Liver Disease. Data for some variables were not available for several cases, so the results presented are based on available information only*.

## Discussion

To overcome increasing organ shortage, the use of extended criteria donors is one of the major solutions. Transplantation of those organs leads to a higher risk of early organ dysfunction, primary non-function, or even graft lost. Therefore, it is of great clinical relevance to assess the status and grade of EAD in clinical practice. However, there is no consensus on the clinical criteria to define EAD.

Early allograft dysfunction created by Olthoff et al. is one of the earliest used definitions containing AST, ALT, INR, and bilirubin, which represent the injury, metabolic, and synthetic functions of the allograft (14). EAD still the most widely used definition because of its simpleness and usefulness for predicting survival prognosis (11, 20–22). Besides, in some machine perfusion randomized controlled trails, EAD was considered as an endpoint (23, 24). However, this dichotomous definition is not able to distinguish severe cases from normal cases. For patients who just meet one criterion like the peak AST/ALT level, they usually have good transplant outcomes. For others with extremely high laboratory indices or meeting more conditions, they might be in a dangerous situation and need special care or even a retransplantation. Besides, there is clearly a difference in graft function between a patient with a pre-transplant bilirubin level of 40 mg/dL whose bilirubin is normalizing, and a patient with liver cancer with a physiological MELD 7 who has a normal pre-transplant bilirubin level and has developed significant cholestasis 1 week following LT. Moreover, Clavien et al. thought that a definition consists of peak transaminases and single value of INR and TBIL in specific time should not be used in perfusion trails (25).

Pareja et al. came up with MEAF to grade and standardize EAD severity (13). Studies had showed that MEAF is related to early graft loss and transplant survival (26, 27). Similar to EAD, some ongoing machine perfusion trials, such as hypothermic oxygenated perfusion and dual hypothermic oxygenated machine perfusion use MEAF as their primary or secondary endpoint (28, 29). Although MEAF uses a continuous concept, it lacks the trend or change rate of variables. Cases with gradually raised laboratory tests after OLT are different from cases with sharply reduced laboratory tests, even though they share similar high tests at the beginning.

Liver graft assessment following transplantation score developed by Agopian et al. is a continuous score composing of AST, INR, TBIL, and PLT within 7 or 10 days. Importantly, it also takes their average, peak, rate of change into account, and uses a cut-off value to discriminate high-risk and low-risk subgroups. Therefore, L-GrAFT might better assess early post-transplant liver graft function. Recently, its prediction power for 3-month graft survival has been validated in both American and European cohorts (15). Importantly, patients in the European cohort were participants from a normothermic machine perfusion trial. Therefore, L-GrAFT risk score might serve as a good surrogate endpoint for graft survival in NMP and other translation studies.

Early allograft failure simplified estimation score was designed by Avolio et al. and had a similar conception to L-GrAFT. They not only thought that post-transplant AST, TBIL, and PLT were important, but also variables concerned with peritransplant and center volume should be noted. So, they were taking MELD score at transplant, number of Packed Red Blood Cell transfused units during surgery, thrombosis and center volume into account, which is reasonable.

The results from the current large single Chinese center report showed that L-GrAFT_7_ risk score had a higher C statistic in predicting 3-month graft or patient survival, compared with MEAF score and EAD. In addition, we found that L-GrAFT_10_, L-GrAFT_7_, and EASE could predict 3-, 6-, or 12-month graft or patient survival equally well. Since the laboratory tests are not performed every day during POD8-10 in our center, L-GrAFT_7_ risk score might be a more feasible choice for assessing early allograft function. In addition, the independent risk factors (laboratory MELD score and CIT) for L-GrAFT_7_ high risk group were identified. Therefore, the results of the current study validated L-GrAFT7 for the first time in a Chinese population.

As for the exclusion of early post-transplant vascular complications (<14 days) cases, similar studies concerning early allograft dysfunction (EAD) have excluded patients with post-operative thrombosis of a hepatic vessel (21, 30). EAD is related to ischemia reperfusion injury, reflecting early dysfunction and poor recovery of liver function in the early-stage post-transplantation. However, hepatic arterial thrombosis (HAT) and portal vein thrombosis (PVT) usually occur within 2 weeks (31). Some studies have shown that the surgical technique is the most important risk factor for HAT and PVT, such as bench arterial reconstruction, reperfusion time, (32) and arterial reconstruction with more than one anastomosis (extra anastomoses) (33). Early post-operative thrombosis of a hepatic vessel is regarded as a non-hepatogenic trigger indirectly leading to the increase of liver enzyme indexes, which will interfere with statistical results of prognosis. Therefore, we excluded those cases.

There are limitations in this study. This single-center study is confined to a relatively limited database and represents a southern China population. A nationwide or even global multicenter study is needed to validate its universality in China or the world. In the future, the design of a new international multicenter prospective study on early allograft failure will be a great help to validate and create a better definition of EAD. Besides, the L-GrAFT_10_ risk score and EASE could not be calculated so perfectly because the laboratory tests were not performed every day during POD8-10 in our center. Cases with not so well recovery tended to have more laboratory tests after a week, which might lead to potential confounding bias. Moreover, 31 cases who underwent a retransplant or died within 10 days were excluded due to insufficient laboratory values to calculate risk scores. The majority of these cases represent an early allograft failure status.

In summary, this large single center study showed that the L-GrAFT_7_ risk score is more capable than MEAF and EAD for predicting short term graft and patient survival. The L-GrAFT_7_ risk score might represent a better definition of EAD and clinical endpoint. Moreover, it is worth to put into use in clinic work or clinical research to evaluate patient outcome in the early post-operation period.

## Data Availability Statement

The datasets presented in this article are not readily available because the original data contains patients individual and private information. Requests to access the datasets should be directed to the correspondence.

## Ethics Statement

The studies involving human participants were reviewed and approved by Independent Ethics Committee for Clinical Research and Animal Trails of the First Affiliated Hospital of Sun Yat-sen University (No. [2020]336). The patients/participants provided their written informed consent to participate in this study.

## Author Contributions

SC, TW, and TL contributed to conception and design of the study. SC, TL, and SH organized the database. SC, TW, and ZJ performed the statistical analysis. SC wrote the first draft of the manuscript. SC, TW, LZ, DW, XZ, ZG, and XH wrote sections of the manuscript. All authors contributed to manuscript revision, read, and approved the submitted version.

## Funding

This work was supported by grants as follows: The National Natural Science Foundation of China [82070670 and 81970564], the Key Clinical Specialty Construction Project of National Health and Family Planning Commission of the People's Republic of China, the Guangdong Provincial Key Laboratory Construction Projection on Organ Donation and Transplant Immunology [2013A061401007 and 2017B030314018], Guangdong Provincial international Cooperation Base of Science and Technology (Organ Transplantation) [2015B050501002], Guangdong Provincial Natural Science Funds for Major Basic Science Culture Project [2015A030308010], Guangdong Provincial Natural Science Funds for Distinguished Young Scholars [2015A030306025], Special support program for training high level talents in Guangdong Province [2015TQ01R168], Science and Technology Program of Guangzhou [201704020150], Science and Technology Program of Guangdong [2020B1111140003], Sun Yat-sen University Young Teacher Key Cultivate Project [17ykzd29], Elite Program specially supported by China Organ Transplantation Development Foundation and Natural Science Foundation of Guangdong Province [2016A030313239].

## Conflict of Interest

The authors declare that the research was conducted in the absence of any commercial or financial relationships that could be construed as a potential conflict of interest.

## Publisher's Note

All claims expressed in this article are solely those of the authors and do not necessarily represent those of their affiliated organizations, or those of the publisher, the editors and the reviewers. Any product that may be evaluated in this article, or claim that may be made by its manufacturer, is not guaranteed or endorsed by the publisher.

## Abbreviations

ALT: alanine aminotransferases
AST: aspartate aminotransferases
AUC: area under curve
AUROC: area under the ROC curve
CIT: cold ischemia time
DBD: donation after brain death
DBCD: donation after brain and cardiac death
DCD: donation after cardiac death
EAD: early allograft dysfunction
EASE: Early Allograft Failure Simplified Estimation
HCC: hepatocellular carcinoma
ICU: intensive care unit
INR: international normalized ratio
L-GrAFT: Liver Graft Assessment Following Transplantation
NMP: normothermic machine perfusion
MEAF: Model for Early Allograft Function
MELD: Model for End-Stage Liver Disease
OLT: orthotopic liver transplantation
PLT: platelet
POD: post operation day
ROC: receiver operating characteristic
TBIL: total bilirubin.
