# Supplementary figures and images for: Prediction of Graft Survival Post-liver Transplantation by L-GrAFT Risk Score Model, EASE Score, MEAF Scoring, and EAD
Source: Front Surg. 2021 Nov 19;8:753056. doi: 10.3389/fsurg.2021.753056 (PMC8641658; doi:10.3389/fsurg.2021.753056)

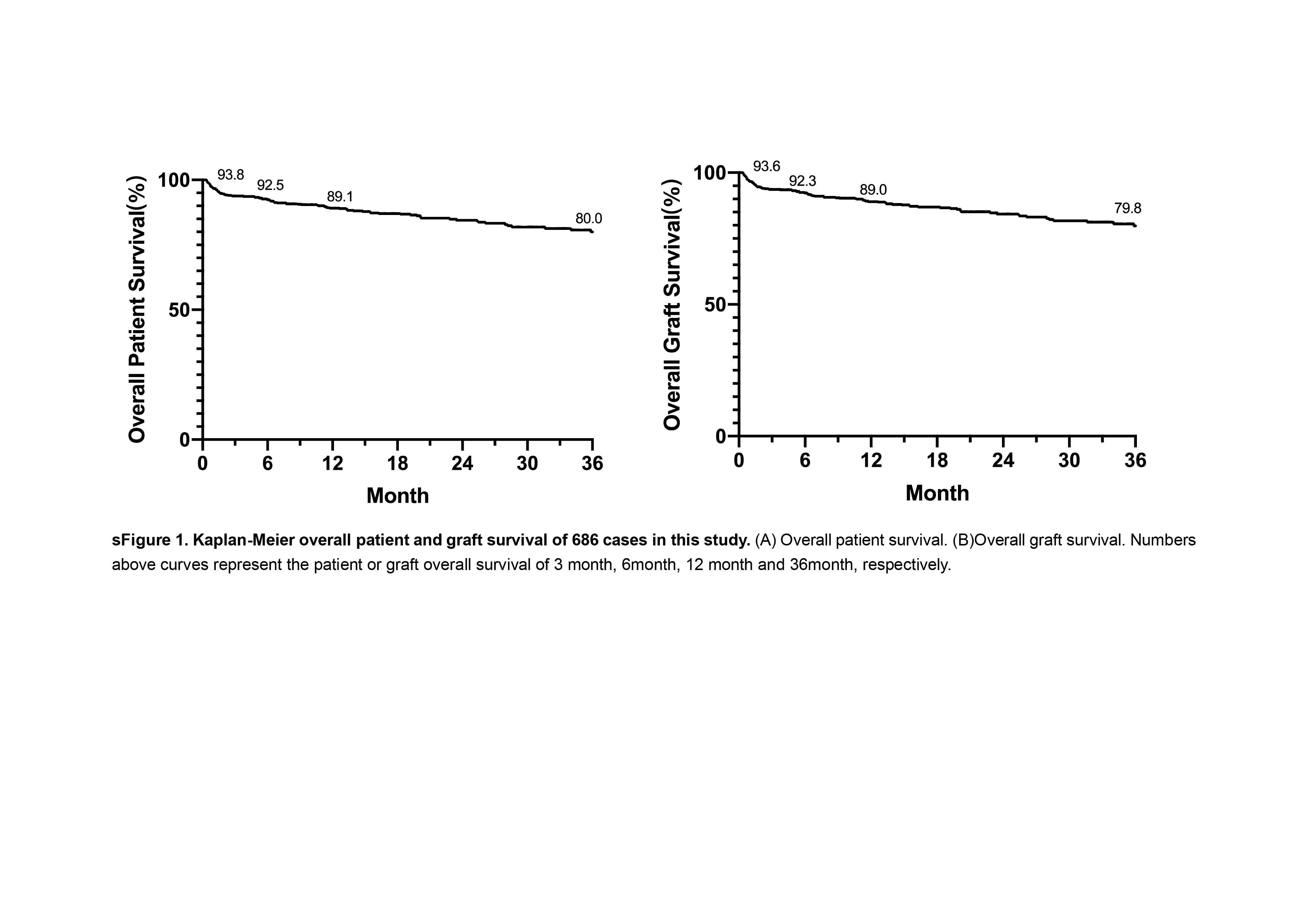

Supplement: Supplementary file 1 [file Image_1.JPEG]

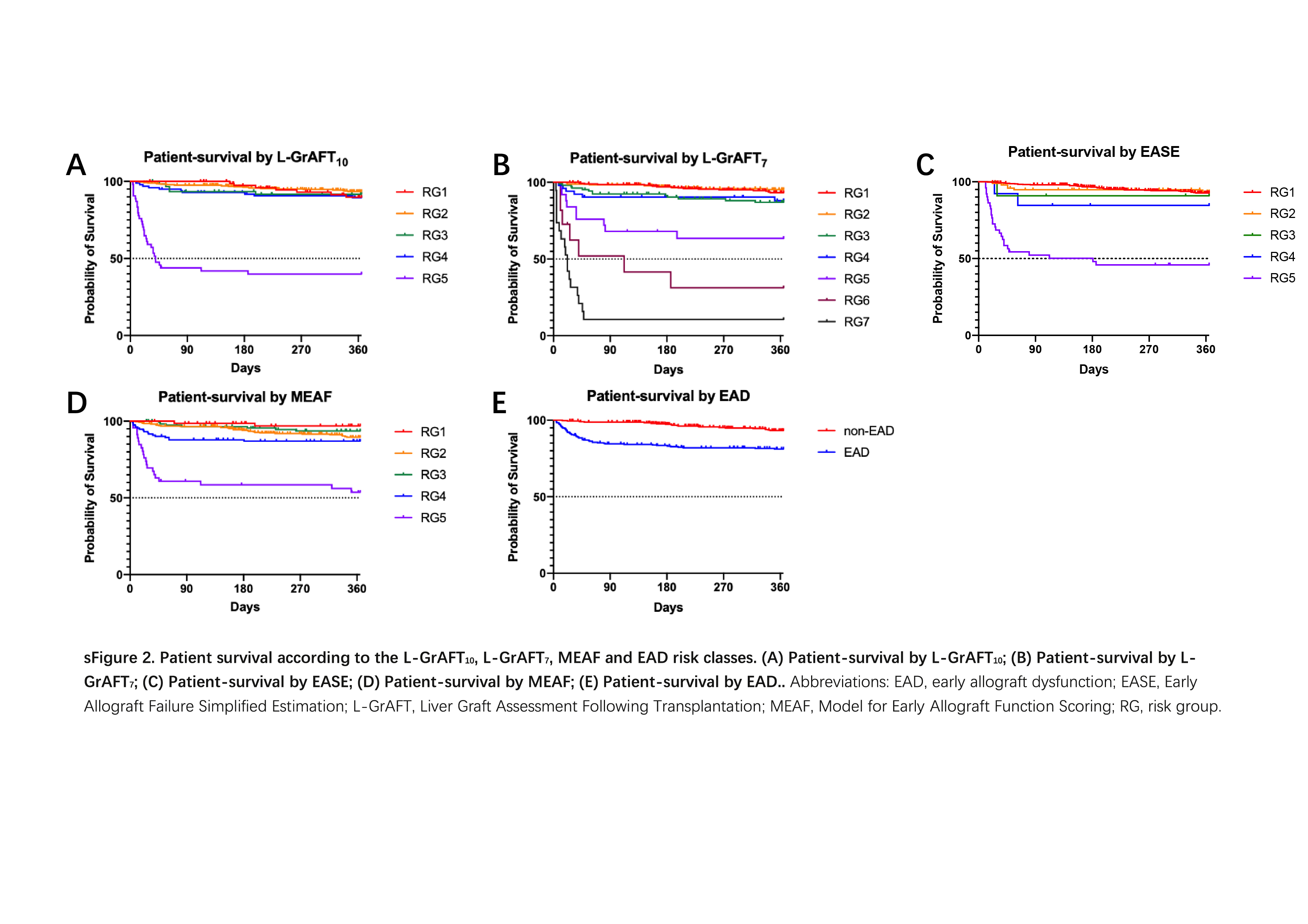

Supplement: Supplementary file 2 [file Image_2.JPEG]
