# Supplementary material for: Prediction of Graft Survival Post-liver Transplantation by L-GrAFT Risk Score Model, EASE Score, MEAF Scoring, and EAD
Source: Front Surg. 2021 Nov 19;8:753056. doi: 10.3389/fsurg.2021.753056 (PMC8641658; doi:10.3389/fsurg.2021.753056)
Supplement: Supplementary file 3 [file Table_1.pdf]

sTable1 Characteristic of 677 orthotopic liver transplantation patients

Variables	Frequency (%) or Median (IQR)
Recipient Demographics	
Age (years)	51 (43, 59)
Gender	
Male	88.8
Female	11.2
Diagnosis	
HCC	53.3
Hepatitis B virus-related cirrhosis	22.0
ACLF	9.7
Alcoholic cirrhosis	4.1
Other	10.8
BMI (kg/m^2)	23.1 (20.8, 24.8)
Height (cm)	169.0 (165.0, 172.0)
Weight (kg)	65.0 (57.5, 71.0)
Comorbidity	
Diabetes	14.8
Hypertension	13.6
Cardiovascular system diseases	3.5
Pretransplantation	
Laboratory MELD score	12 (7, 22)
CREA (μmol/L)	71 (59, 89)
TBIL (μmol/L)	43.4 (20.1, 229.0)
INR	1.34 (1.12, 1.89)
Laboratory MELD score (HCC)	8 (5, 13)
Laboratory MELD score (Others)	18 (11, 28)
Child-Pugh score	8 (6, 10)
Infection	10.5
Renal replacement therapy	1.2
Mechanical ventilation	1.2
Donor	
Age (years), median	38 (24, 47)
Gender	
Male	74.9
Female	25.1
BMI (kg/m^2)	22.1 (20.3, 23.9)
Height (cm)	168.0 (160.0, 170.0)
Weight (kg)	60.0 (55.0, 68.0)
Cause of death	
Trauma	47.7
CVA	37.2
HIE	8.1
Others	6.9

The Chinese Classification of Deceased Organ Donation	
C-I (DBD)	79.3
C-II (DCD)	16.2
C-III (DBCD)	4.4
Comorbidity	
Hypertension	10.1
Diabetes	9.3
Cardiovascular system diseases	0.9
DRI	1.66 (1.41, 2.06)
Peri-operation	
Anhepatic phase (min)	54 (43, 65)
WIT (min)*	5 (6, 10)
CIT (min)	421 (334, 522)
Total operation time	450 (390, 525)
Transfusion	
RBC (unit)	5.0 (2.3, 8.0)
Fresh frozen plasma (unit)	7.5 (5.0, 10.6)
Intraoperative hemorrhage (ml)	1500 (1000, 2800)
Post-operative	
Mechanical ventilation (hour)	17.5 (12.0, 43.0)
ICU stays (hour)	40.0 (23.0, 87.6)

Abbreviations: ACLF, acute-on-chronic liver failure; BMI, body mass index; CIT, cold ischemia time; CREA, creatinine; CVA, cerebrovascular accident; DBCD, donation after brain and cardiac death; DBD, donation after brain death; DCD, donation after cardiac death; DRI, donor risk index; HCC, hepatocellular carcinoma; HIE, hypoxic-ischemic encephalopathy; ICU, intensive care unit; INR, international normalized ratio; MELD, Model for End-Stage Liver Disease; TBIL, total bilirubin; WIT, warm ischemia time.

Note: Data for some variables were not available for some recipients or donors, so the results presented are based on available information only.

*Only be calculated In DCD and DBCD cases.
